# A systematic review of geographic indices of disadvantage with implications for older adults

**DOI:** 10.1172/jci.insight.141664

**Published:** 2021-10-22

**Authors:** William R. Buckingham, Lauren Bishop, Christopher Hooper-Lane, Brittany Anderson, Jessica Wolfson, Stephanie Shelton, Amy J.H. Kind

**Affiliations:** 1 School of Medicine and Public Health, Department of Medicine, Division of Geriatrics,; 2 School of Social Work and Waisman Center, and; 3 Ebling Library, University of Wisconsin-Madison, Madison, Wisconsin, USA.; 4 VA Geriatrics Research and Education Clinical Center (GRECC), Madison VA Hospital, Madison, Wisconsin, USA.

**Keywords:** Aging, Epidemiology

## Abstract

**BACKGROUND:**

Neighborhood-level socioeconomic disadvantage has wide-ranging impacts on health outcomes, particularly in older adults. Although indices of disadvantage are a widely used tool, research conducted to date has not codified a set of standard variables that should be included in these indices for the United States. The objective of this study was to conduct a systematic review of literature describing the construction of geographic indices of neighborhood-level disadvantage and to summarize and distill the key variables included in these indices. We also sought to demonstrate the utility of these indices for understanding neighborhood-level disadvantage in older adults.

**METHODS:**

We conducted a systematic review of existing indices in the English-language literature.

**RESULTS:**

We identified 6021 articles, of which 130 met final study inclusion criteria. Our review identified 7 core domains across the surveyed papers, including income, education, housing, employment, neighborhood structure, demographic makeup, and health. Although not universally present, the most prevalent variables included in these indices were education and employment.

**CONCLUSION:**

Identifying these 7 core domains is a key finding of this review. These domains should be considered for inclusion in future neighborhood-level disadvantage indices, and at least 5 domains are recommended to improve the strength of the resulting index. Targeting specific domains offers a path forward toward the construction of a new US-specific index of neighborhood disadvantage with health policy applications. Such an index will be especially useful for characterizing the life-course impact of lived disadvantage in older adults.

## Introduction

Neighborhood-level disadvantage shapes the socioeconomic status of individuals and influences individual behavior and outcomes through the shared social, service, and physical environments of their communities (1). The life-course impacts of neighborhood-level disadvantage on health outcomes may become particularly apparent in older adults. In older adults, studies have shown that increasing chronic pain (2), increasing cognitive decline (3), and increased risk of falls (4) are just a few examples of the impacts, explained in part by increased neighborhood-level disadvantage.

Neighborhoods that are characterized by high neighborhood disadvantage typically have high rates of poverty as well as other markers of poverty, such as high rates of single-parent or non-parent (often grandparent headed) households, low rates of higher education attainment, and low rates of home ownership. This is particularly problematic for older adults who are more likely than younger adults to spend large amounts of time within their neighborhood and to rely on local social structures for support. Older adults often have lessening mobility, causing their lived area to shrink over time as they stay close to their residence (5). Further, long-term exposure to stressors like disadvantage can have deleterious effects on health, and these health impacts of stress and disadvantage can be present in older adults (6). Multiple studies, including seminal work like the Moving to Opportunity Study, have found that the relative level of neighborhood disadvantage is an important predictor of health outcomes across the life course (7). Older adults can see increased cognitive decline, decreased physical ability, and heightened concern for their safety when exposed to living in a highly disadvantaged neighborhood.

One way to measure neighborhood disadvantage is through the use of indices at small units of geography (i.e., the block group level). Such indices have the potential to measure nuance within larger geographic contexts. For instance, in some cities, neighborhoods with high levels of advantage abut neighborhoods with high levels of disadvantage, and these nuanced differences may matter for health, yet may be missed when using larger geographic contexts, which may mask these variations (8).

Predominantly constructed using national census figures, indices of neighborhood-level socioeconomic disadvantage are employed by governmental agencies outside of the United States to identify and target high-needs populations. The use of indices of disadvantage to describe the health of a population at small units of geography is a practice that has been undertaken for decades, especially in a European context (9–11). However, indices specifically targeted to older adults are absent within the findings in this study, so expanding the study to indices in general allows for a comprehensive capture of the character of indices of deprivation.

Despite the potential for translation difficulties between the United States and other contexts, the use of census data allows for the possibility of using data elements that are similar between these contexts, making a worldwide approach useful for informing US index creation. Within the United States, there has been no coordinated federal effort to understand neighborhood-level disadvantage. One recent development that has emerged to address small-area disadvantage in the United States is the newly released Neighborhood Atlas, a web-based dissemination tool for geographic-based metrics of disadvantage, including the 2018 Area Deprivation Index (ADI) (12). The ADI provides national census block group–level area-based disadvantage classifications derived from data from the US Census Bureau’s American Community Survey (ACS).

Disadvantage indices within the US context have demonstrated a lack of consensus about how to best measure neighborhood disadvantage in the United States. A US-specific index that quantifies the relative level of neighborhood disadvantage and that is predictive of health outcomes in older adults would be of high utility for research and policy because it could be used to target and test interventions at both the individual and neighborhood level, as well as to determine distribution of resources to combat disadvantage. In an age of big data availability, indices of neighborhood disadvantage have new and potentially transformative uses within health care and policy settings. The ADI may be such an index.

However, in order to begin the process of establishing a single index for the United States, a full review of the existing state of indices is necessary. To fill this need, we conducted a systematic review of indices in the English-language literature in order to begin to understand the lived disadvantage of older adults, to inform policy, to guide decision making about the distribution of resources to neighborhoods, and ultimately, to provide new tools for incorporating this understanding into health policy. Within this review we aim to (1) summarize the complete suite of variables used in the construction of indices of neighborhood disadvantage; (2) distill these variables into functional groupings; and (3) summarize the main components needed to construct an index of neighborhood disadvantage that could be used within the US context. An additional aim was to identify domains commonly used in indices of disadvantage for the purpose of informing future development of US-specific metrics.

## Results

**Figure 1:**
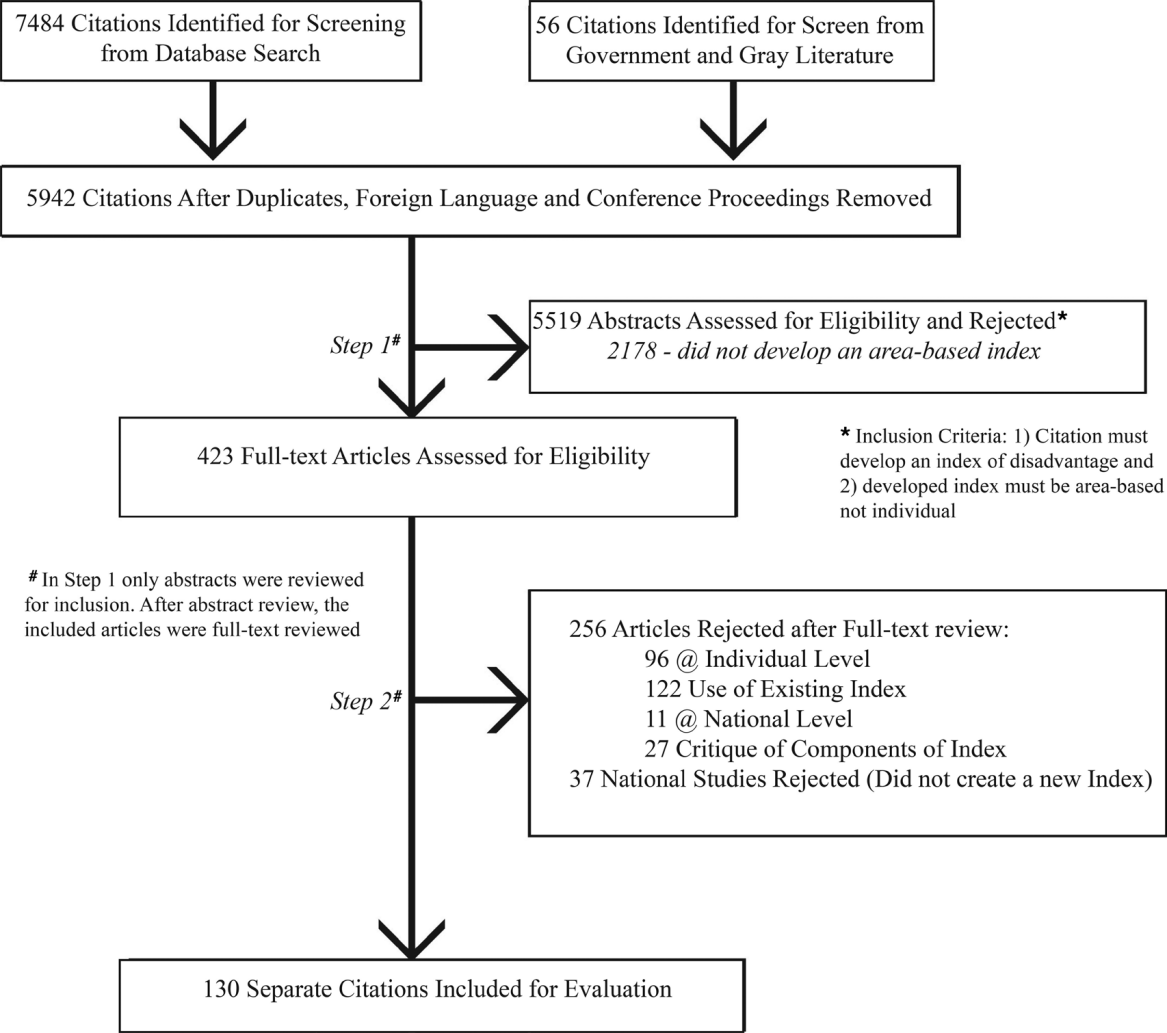
Article selection flowchart.

Our initial database search revealed a total of 6021 unique articles that were included for initial review. Of the 6021 abstracts included in the database queries, 407 met the first-stage inclusion criteria of constructing an index of neighborhood disadvantage. Each of the first-stage papers was then evaluated in its entirety to determine the type of index being created. Of the 407 papers, 130 met the final inclusion criteria of requiring construction of an area-based index (index of neighborhood disadvantage) and were selected for complete evaluation and review. The papers not selected ranged from critiquing indices, to constructing individual level indices, to using existing indices, as shown in Figure 1. A full list of these papers is provided in a supplemental reference list (see Supplemental Table 1; supplemental material available online with this article; https://doi.org/10.1172/jci.insight.141664DS1).

### Scientific and geographic context.

The indices evaluated came from 2 distinct literature types: traditional academic papers (95.4%) and government white papers (4.6%). The functional construction of the indices did not differ significantly between the academic and government papers, but the goals were distinct. The government white papers were written to share the final index construction methods used in a national context. The research papers took 1 of 2 tracks: they attempted to create a general index and describe the construction of said index, some of which led to the construction of the national indices, as in the case of the Scottish Index of Multiple Deprivation (SIMD) (Carstairs) and the Indices of Multiple Deprivation (IMD) (Townsend) (9, 11). Others sought to develop an index with a goal-oriented approach and would apply the index for the purpose of understanding the research domain in question (13–16). Ultimately, the 2 origin points resulted in very similar methods of actual index construction.

Geographically, 23.8% of the papers (all academic) come from a US context; 38.5% (90% academic) of papers, including the well-known indices by Carstairs (9), Jarmin (10), and Townsend (11), come from the European Union and 37.7% (98% academic) come from countries outside of the European Union and United States (including New Zealand, Australia, Brazil, and Canada).

### Statistical methods.

The most common statistical methods used to construct area-based indices were principal components analysis and factor analysis. Within these methods, multiple variable transformations and rotations were used (e.g., exponential, logistic, arcsine); 37 (28.5%) studies used factor analysis and 61 (46.9%) used principal components, for a total of 98 studies or 75.4%. The others employed a variety of other methodologies.

### Geographic scale.

The geographic scale of the included studies varied by national setting. The scales ranged from small (census block groups and tracts — roughly 1200 and 4000 people, respectively, defined by the US Census Bureau) to units that were quite large (zip codes, counties, and municipalities). Geographic scales in a US context (ordered by prevalence of representation within studies reviewed) included the zip code (7 occurrences), the county (8 occurrences), the census block group (9 occurrences), municipality (17 occurrences), and census tract (32 occurrences). European contexts included “small areas” (7 occurrences), districts (13 occurrences), and a number of “defined areas” (34 occurrences), which varied tremendously in their operationalization among studies. The geographic scale used in the construction of each index was also affected by the pragmatic considerations, such as availability of data. For instance, where only highly suppressed or large-scale data were available, county, municipality, zip code, or district-level geographic scale were used. When more detailed small-area data were available, block group (i.e., neighborhood) geographic scale was used. These smaller-scale areas were more common in the most used indices in Europe (Carstairs, ref. 9; Jarmin, ref. 10; Townsend, ref. 11; SIMD, ref. 17; IMD, ref. 18). Further, indices using small-scale units offered the most granular detail and were found to be the most widely applied in practical settings (all outside the United States).

The description of geographic data across studies had a lack of standardization in that geographic terms were conflated (i.e., census block tract combined 2 geographic levels) or improperly described when detailing the areal unit of analysis. These vague or misnamed terms were treated as a new and independent geography. Although these instances were small in number, no attempt was made to divine the intent of the authors to avoid introducing uncertainty. For the purpose of the systematic review, an accurate count of named geographies was determined to be the appropriate method, without translation.

### Domains.

Education and employment were the most prevalent variable domains in the literature, as shown in Supplemental Table 1, with 80.0% (*n* = 104) and 83.9% (*n* = 109) of the indices including a variable from those domains, respectively. Educational attainment level, 75.4% (*n* = 98), and unemployment, 78.5% (*n* = 102), were the most prevalent subdomains in the literature. Neighborhood structure (a broad unit, including built environment, safety, and social structure; 79.2% for *n* = 103) and personal housing and housing economics (*n* = 80 for 61.5%) were the next most common. Other variable domains identified included income (59.2% for *n* = 77), demographic makeup (*n* = 47 for 36.2%), and health (*n* = 23 for 17.7%). Combinations of domains were possible, although rare, and in those cases, the classification was with the dominant domain (for example, percentage of 25 and older by high school and above and white-collar occupation would be placed in the education domain as the primary delineator). In many cases, multiple variables within a domain were included in the index construction.

The subdomains shown in Table 1 represent the major thematic domains contained within the larger domains. These domains and subdomains cover the majority of possible topics. Some of these domains are likely geographically dependent; for example, crowded housing is likely to be skewed to urban geographies. Similarly, some housing quality measures, such as internal running water and housing value, will be geographically heterogenous and potentially skewed by the urban or rural makeup of the location.

The domains and subdomains were formed by summarizing the complete list of variables used in these indices. These domains appear stable across all of the surveyed indices. Although a single domain does not appear in every index, all of the indices contained a subset of the domains. It is difficult to determine a universal rule for index creations as to the number of domains to be included. However, the median number of domains across all studies is 5, but the most successful indices use 5, 6, or 7 domains. Indices with between 5 and 7 domains strike the balance between feasibility of data collection and enough specificity in constructs measured to measure meaningful differences in neighborhood disadvantage and might serve as a guide for future studies that develop and/or validate indices of neighborhood disadvantage. For a universal index, it is likely that 5–7 domains should appear, but more targeted indices may only look at a single domain like an index of educational attainment.

## Discussion

Core indicators of neighborhood disadvantage identified by this review include (a) education, (b) income, (c) personal housing and housing economics, (d) employment, (e) neighborhood structure, (f) demographic makeup, and (g) health. Table 1 shows a mapping of the ACS to these domains. Of these core indicators, all but health have direct correlates that are universally accessible at small units of geography. Although health measures are available at larger areas, small-area health estimates are currently lacking and make this domain the only large domain with no direct national proxy in a US context. These core indicators offer the overarching themes that make up the majority of indices in some combination. By distilling the variables into these 7 core indicators, subsequent index development can be constructed within a framework established by the rich body of research on index construction. Other domains around sexual identity, indigenous health, disability status, and potentially rural or urban status (among possible others) have the potential to add to indices, but these have not been identified within any of the studied indices.

The literature surveyed extends beyond the traditional academic literature into governmental reports given that these indices have been adopted by a number of governments (e.g., England, Scotland, ref. 17; Wales, ref. 19; New Zealand, ref. 20) to understand and improve the health of constituents by identifying and targeting areas of high need. Of the 130 papers that met inclusion criteria, 76.1% (*n* = 99) were from non-US contexts. Although the non-US indices are afforded data opportunities unavailable in a US context and differences in national data collection and the structural etiologies of population-specific disadvantage may differ across contexts, the social experience of disadvantage continues to primarily be driven by differences in allocation of the social determinants of health, regardless of context. Another way of stating this is that the core domains identified above are consistent across national boundaries, yet the reasons for unequal distribution of social determinants of health may differ. In the United States, structural racism may have originally led to the unfair and unequal allocation of social determinants of health, resulting in neighborhood disadvantage within a particular region, particularly in the case of redlining of certain urban neighborhoods. Further, while identical variables may not exist, similar themes can be found within most of these national contexts, making the domain analysis performed here more applicable generally. Distilling disadvantage into comparable domains allows for each national index to emphasize elements that are of particular importance — for example, the demographic element may be particularly important in a US context given issues of past and current structural racism in the United States, but it may be less important within a European context. Each index may incorporate (or not) the domains, although the weights likely differ. Current US indices are few in number and often specialized, but do provide a baseline for future expansion of this work.

The indices reviewed did not specifically target any age, gender, or racial group. Each index was a general description of the total population. However, a large body of literature indicates that neighborhood disadvantage has been linked to problematic health outcomes in older adults. Disability prevalence (21), cognitive function (22), subjective appraisals of health (23), and mental health (24) are just some of the health concerns that increase in older adults when they live in disadvantaged areas. Indeed, older adulthood is the life stage during which the impact of disadvantage on morbidity and mortality is likely to become particularly pronounced. The forms of disadvantage cited include income and education disadvantage within the lived area, mirroring the more general indices of disadvantage. This concurrence appears to suggest that understanding the overall structural disadvantage of an area has important effects on the health of older adults — making the case that understanding the components of neighborhood disadvantage across the literature is critical to address the disadvantages faced by the older adult population. For example, areas of high disadvantage can make accessing health care resources more difficult because highly disadvantaged areas exhibit greater transportation deficiencies as well as income shortages. Although the indices reviewed are not specifically created with the older adult as the focus, the outcomes are measures that pertain to older adults in very specific domains.

Calls for government adoption of the effort to standardize an index of disadvantage for the United States have been made in the literature (25). National census data, including the ACS, offer the easiest and most comprehensive means of understanding the income, education, employment, and housing variables within small areas of geography in the United States, as shown in Table 1. Within a US context, the ACS provides data for the majority of identified domains across the United States at precise geographic levels. The yearly refresh of the ACS estimates is also a distinct strength, allowing for a US-based index to maintain a temporal update. However, ACS variables have larger margins of error than the previous long-form census data. Further, for small areas, the ACS is only offered as a 5-year moving average, making direct point-in-time estimates diluted with respect to large economic or housing shocks. Opportunities to improve the ACS are present, but for now, it is the best US data source available at geographically discrete levels.

However, ACS data are missing important theoretical factors, such as sexual orientation, disability status, and health care availability (26). Increasing the availability and quality of such data would enable future indices to target components that could increase the power of these indices. Furthermore, ACS and US Census data may have undercounts of certain disadvantaged populations (27). This undercount creates both a selection and a measurement bias in terms of the originating data and the extent to which certain variables are measured and included in the indices. These types of bias are inherent in the use of census-based data and are a concern to be considered when developing new indices, especially when looking to expand beyond census data to new sources of information. Unfortunately, these types of bias are difficult to quantify precisely and mitigate accurately. Attempts to do so potentially introduce further error into the measurements. Therefore, this limitation is one to be noted and acknowledged in any index based on census data or a census product, such as ACS.

Despite these issues, the ACS does offer the best small-area data at the present time. However, adding data outside of the ACS would be a logical step for any new index. This would potentially overcome the issues within the ACS in terms of sample size and undercount of vulnerable populations. Supplementing with data from a source such as Medicare (the only universal health insurance program for a specific age group in the United States) would allow US-based indices to expand their inputs beyond the typical census-based statistics currently in use and incorporate neighborhood-level disadvantage health data specific to older adults. The challenge to incorporating non–census-based data into any index is the geographic mismatch between the index and the potential input data that renders the different data incompatible for an index. This technical challenge could be overcome but would require considerable resources and effort. Further, any new input data would either need to provide universal coverage of the population (similar to the census) or would have to directly reflect the population of interest (potentially Medicare as a proxy for an index for older adults). Additionally, the inclusion of basic demographics has the potential to bias the index and remove modeling power. Cautious testing would be needed prior to taking such a step.

By using census data as the primary means of constructing these indices, data availability is a driver of the final index rather than a theoretically grounded approach. Often census data are missing factors, such as sexual orientation, disability status, and in the United States, health system differences based on health care availability, and these missing factors limit the indices’ ability to reflect the entire population. The literature has shown that these diverse contexts are underrepresented. Increasing the quality of data available on diverse health disparities would enable future indices to target the components that might have the most impact on decreasing disparities in diverse contexts. Ultimately, the components of interest are likely to vary between contexts, but a modular build for future indices may offer the flexibility needed to address the unique perspectives needed.

US-based indices created with data specific to older adults have the potential to capture (a) the health of the full population given the universality of Medicare and (b) the impact of neighborhood disadvantage on health in an age group for whom health disparities are likely to be more pronounced. These types of targeted indices may arise from subsets of the larger index similar to the English IMD. The European IMD have a 30-year track record of providing neighborhood-level disadvantage metrics that are effectively used to target disparities both generally and in a targeted manner with the domains of the indices. Additionally, more in-depth geographic data analysis could further enhance a newly created, US-specific index of neighborhood disadvantage, through analysis of community-level variables that are linked to the core indicators of neighborhood disadvantage identified by this review.

Despite comprehensively searching and analyzing over 6000 articles and government reports, there are some limitations to this review. First, the search strategy was limited to academic and public-sector reports that were electronically searchable and to English-language publications. Although this may have limited the number of studies included in the final review, the concordance between findings related to our 7 identified core indicators of neighborhood disadvantage suggests that additional literature would have been unlikely to substantively alter findings. Second, we focused exclusively on area-based indices at the expense of individual-level metrics. Adults living in low-education areas fare less well regardless of their individual characteristics with respect to cognitive function; however, cardiovascular disease and functional status attenuate the association with neighborhood disadvantage in the individual. The issue of individual factors versus neighborhood context is relevant, but does not diminish the need to understand the context that a general index of disadvantage can provide. Finally, when extrapolating to future index development, it is possible that the survey of indices has missed variables and domains that may be important to include in a future index. Additional research on this area is needed.

The opportunity to develop new US-based indices of disadvantage offers a wide variety of implications. Such work could contribute to a new array of diverse metrics that can be offered on public platforms, such as the Neighborhood Atlas, which provides an update to the ADI, to catalyze new research, policy, and intervention efforts (10). This review provides a clear foundation upon which subsequent indices can determine the domains and variables for inclusion. We hope that this review will serve as a launching point for these next steps and for novel metric development.

Research has shown that socioeconomic disadvantage has deleterious effects in older adult populations, from cognitive decline to increased chronic pain. The development of new, robust indices of neighborhood disadvantage that are applicable within the US context and have direct implications for the health of older adults is the ultimate goal of this research. A comprehensive systematic literature review like this is a crucial first step toward that goal. The 7 core indicators of neighborhood disadvantage identified by this review — education, income, personal housing and housing economics, employment, neighborhood structure, demographic makeup, and health — are critical domains to potentially include in the development of new indices of neighborhood disadvantage. These domains provide a better understanding of the environments within which older adults live. Cumulative community-level impact has direct consequences on the lived experience of older adults. Using the identified domains to describe the community in which older adults live is a critical step to understanding disadvantage in older adults and toward development of better interventions to improve health in older adults.

Indices of disadvantage have long played a central role in understanding of health in non-US contexts. In the United States, these indices have been specialized and have not looked at a general framework for their construction nor specifically to how to construct an index to understand disadvantage in the older adult population. By summarizing indices to the major components — income, education, personal housing and housing economics, employment, neighborhood structure, demographic makeup, and health, this systematic review has established a framework that future index development can build upon. It is clear that new indices for the United States offer the opportunity to construct metrics that will aid in the understanding of the ways in which lived disadvantage affects older adults’ health outcomes and health outcomes for the general populace as well. The ADI as currently conceived (with 5 of the 7 identified domains) is a strong starting point for the continued development and refinement of a US-based index.

## Methods

### Search strategy.

We constructed a detailed search strategy to fully capture the academic and government literature on indices of neighborhood disadvantage with the help of a reference librarian who specializes in systematic review methodology. We searched the Scopus, SocIndex, and Sociological Abstracts databases for articles that met our search strategy of English-language papers from 1970 to the present. Scopus includes 100% of the journal titles covered in MEDLINE (PubMed), EMBASE, and Compendex, as well as several other social science and humanities resources. Base terms of “socioeconomic”, “disadvantage”, “disparity”, “deprivation”, and “underprivileged” were combined with “index” or “indices” in the first step. Additional terms to describe geographic units were added to broaden the search and were combined with the original search and with the broadening terms, including “score”, “measure*”, “assess*”, and “identif*”. SocIndex and Scopus were searched using the multifaceted strategy. We used only the first step of our search strategy for Sociological Abstracts because of the number of duplicates with the results from SocIndex and Scopus. An Endnote library including the literature citations and abstracts to assist in screening results for initial inclusion in the systematic review was created from the results. Duplicates, conference proceedings, and non–English-language publications were excluded. References were not screened for additional studies to include. Further, article quality was not assessed because this review only sought to identify the variables and domains present in published indices, rather than offer critique of the resulting index. Choosing not to screen for quality ensured that we did not reduce the pool of papers screened. Although this potentially allowed indices of questionable quality to be included, the choice of variables and structure of these indices is still informative in the overall understanding of how to approach indices of disadvantage.

In addition, government-sponsored index development was captured through references in the indices captured in the literature search and through selection of commonly cited national indices. The inclusion of government indices and gray literature in this study is a significant departure from the typical systematic review methodology and offers a more robust set of findings for this topic. Government index development is a productive area both in the development of indices and in their application to problems of public health, and an omission of this literature would leave an unacceptable gap in the analysis.

### Inclusion criteria.

The PRISMA flowchart of paper selection is shown in Figure 1. Articles were selected for inclusion in this review if they featured the development of an index of neighborhood disadvantage and the developed index was area-based, as opposed to an individual- or household-level index.

After the initial gathering of academic and government literature, the first stage of inclusion screening involved abstract review to evaluate whether studies created, rather than merely used, an index. Studies that used an established index of neighborhood disadvantage but did not construct an index did not meet the inclusion criteria for this review. In instances where the abstract was insufficient to determine whether the study met this initial inclusion criterion, we briefly reviewed the full text of the article. Articles flagged as potentially creating indices were evaluated before advancing to the final stage.

In the final stage, each paper was evaluated to determine the type of index being constructed (e.g., neighborhood or individual). During the full article review, if it was determined that the index being created was at the national level or at the individual (or household) level, then the study was rejected. Further, articles providing only critique of an index or using an index — but not creating an index — were rejected. At least 2 authors evaluated each paper, and those that met the inclusion criteria were moved into the data extraction phase. In the case of disagreement, the lead author was included and made the final decision about inclusion. Agreement was not measured during the screening efforts. Additionally, the quality of the paper was not evaluated in this (or subsequent) stages because the development or lack thereof of an index was informative.

### Data extraction.

We then extracted data about the constructed indices from all papers that met inclusion criteria. These included (a) statistical methodology, (b) geographic scale, (c) variable choice, and (d) inclusion domain. After extraction, index variables were grouped by similar topical area. After grouping was complete, the groups were evaluated to determine the overarching domain for classification purposes and to distill the variables into a categorical framework. In practice, this meant for the education domain, variables such as population with a bachelor of arts degree, population without a high school degree, and literacy would all be grouped together and ultimately classified as within the education domain. This process involved multiple reviewers to ensure agreement in the variable placement, with the lead author mediating any disagreement in the process.

### Included variables and domains.

During the analysis of the papers selected for inclusion in this review, 1353 variables were captured, of which 854 were unique or identified as distinct. The variables were then grouped into subdomains and larger variable domains based on similarity; for example, all education variables were clustered into an education domain despite a disparate number of education-specific variables across the study. Commonalities in variables exist despite national census differences — these commonalities are reflected by the similarities in variable definitions between the national census instruments (for example, percentage unemployed, unemployment rate, proportion unemployed).

Domains were determined by evaluating each individual variable and placing it in a corresponding domain group. Indices could and often do contain multiple elements within the same domain. For example, the English IMD includes rates of theft, criminal damage, violence, and burglary, all within the crime domain (18). Ultimately, the variable list was summarized to 7 domains, each varying in its prevalence within each index. Although these domains come from a variety of national indices, the universal similarities in the summarized domains allowed for the application across national boundaries. Subdomains were also constructed to allow the nuance within each domain to appear. Construction of the domains provides a framework of relevant information that should be considered when moving into index construction. Domain assignments were evaluated and adapted by a multidisciplinary team of 11 members, made up of physicians, social workers, nurses, and speech language pathologists. This multidisciplinary validation step ensured the variable domains were rigorously chosen and evaluated. The final variable domains constructed from the universe of variables can be seen in Table 1.

## Author contributions

WRB designed the review and was the primary author. LB was the secondary author and contributed to the review. CHL designed the searches and collected the pool of articles. BA, JW, and SS reviewed the abstracts and classified the full pool of articles for inclusion. AJHK obtained funding, provided the overarching guidance, and reviewed the paper at each stage of development.

## Supplementary Material

Supplemental data

Trial reporting checklists

ICMJE disclosure forms

## Figures and Tables

**Table 1:**
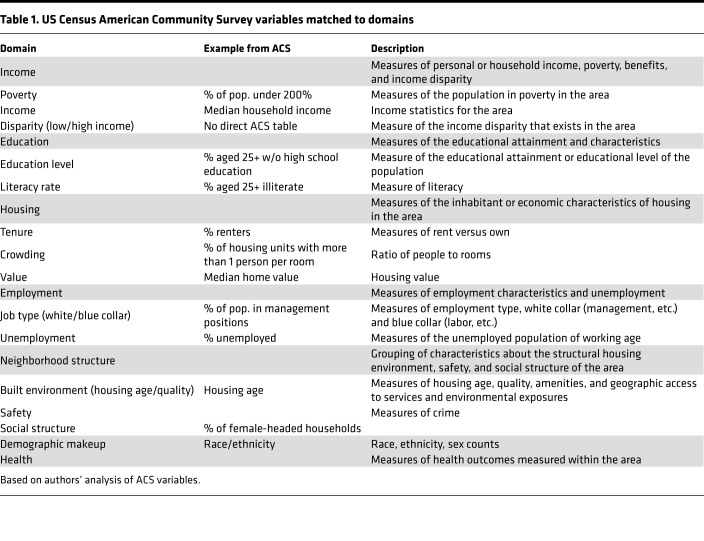
US Census American Community Survey variables matched to domains

## Version 1 09/21/2021

In-Press Preview

## Version 2 10/22/2021

Electronic publication
