# Supplementary material for: A systematic review of geographic indices of disadvantage with implications for older adults
Source: JCI Insight. 2021 Oct 22;6(20):e141664. doi: 10.1172/jci.insight.141664 (PMC8564893; doi:10.1172/jci.insight.141664)
Supplement: Supplemental data [file jciinsight-6-141664-s071.pdf]

Supplemental Table 1

Index	Reference	Country	Scale	Stats	INCOME	I-Poverty	I-Income	I-Disparity	EDUCATION	ED-Level	ED-Literacy	HOUSING	H-Tenure	H-Crowding	H-Value	EMPLOYMENT	EM-Job Type	EM-Unemp	NBHD STRUCTURE	NS-Built Env	NS-Safety	NS-Soc Structure	DEMOGRAPHICS	HEALTH	Variable List
1	Atkinson, J., Salmond, C., & Crampton, P. (2014). NZDep2013 Index of Deprivation. Dunedin: University of Otago.	NZ	small areas	FA	x	x		x	x	x		x	x			x		x	x	x		x			People aged <65 with no access to the Internet at home; People aged 18-64 receiving a means-tested benefit (welfare); People living in households with equivalised income below an income threshold; People aged 18-64 unemployed (over course of a week); People aged 18-64 without any qualifications; People not living in own home; People aged <65 living in single parent family with dependent children; People living in households below an equivalised bedroom occupancy threshold, AKA overcrowding; People with no access to a car; People with no access to any phone at home
2	Rajekal, M., Jan, S., & Jarman, B. (1996). The Swedish UFA score: An administrative tool for identification of underprivileged areas. Scandinavian Journal of Social Medicine, 24(3), 177-184.	SWE	Small Area Marked Statistics	Z-scores								x		x		x	x	x	x			x	x		Elderly Alone; Under 5; Single Parent; Unskilled; Unemployed; Overcrowded; Moved; Foreigners
3	Bauer, J., Brueggmann, D., Ohlendorf, D., & Groneberg, D. A. (2016). General practitioners in German metropolitan areas - Distribution patterns and their relationship with area level measures of the socioeconomic status. BMC Health Services Research, 16(1).	GER	Metros	PCA	x	x										x		x	x	x		x	x	x	Pop Dens; Distance; 65+ per 100 (15-64); Migrants; Household Size; Employment Quota; State Subsidy; Owne Cards; Mortality
4	Bell, N., & Hayes, M. V. (2012). The Vancouver Area Neighbourhood Deprivation Index (VANDIX): A census-based tool for assessing small-area variations in health status. Canadian Journal of Public Health, 103(SUPPL.2), S28-S32.	CAN	Census Dissemination Areas and Loacal health areas	stdized z-scores	x		x		x	x		x	x			x		x	x			x			No High School; University; Single Parent; Ave Income; Home Ownership; Employment ratio
5	Benach, J., & Yanai, Y. (1999). Geographical patterns of excess mortality in Spain explained by two indices of deprivation. Journal of Epidemiology and Community Health, 53(7), 423-431.	ESP	small areas	FA					x		x	x		x		x		x	x			x			unemployment (percentage of unemployed in the population aged 15 to 64 years); illiteracy (percentage of illiterate people in the population over age 10); social class (number of persons with unskilled occupations divided by all people who ever worked); overcrowding (percentage of households with more than one person per room)
6	Beznaczko, N. (2003). Breakthrough Measuring Neighborhoods. Journal of Applied Measurement, 4(2), 137-152.	USA	community areas		x	x	x		x	x						x		x	x		x	x		x	female-headed families; male joblessness; welfare dependency; high graduation; percentage of residents with earned income less than federal poverty threshold; median household income; deaths of infants less than one year of age per 1,000 live births; homicides and suicides per 100,000 population
7	Bonfim, C., Alves, A., Costa, T. R., Alencar, F., Pedroza, D., Portugal, J. L., & Medeiros, Z. (2011). Spatial analysis and privation index to identify urban areas with a high risk of lymphatic filariasis. Tropical Medicine and International Health, 16(6), 748-755.	BRA	census tract	pca	x	x			x	x		x	x	x					x	x					proportion of households with inadequate water supply; proportion of households with inadequate sewage sanitary; proportion of households with inadequate solid waste collection; proportion of heads of households with an income of between 0.5 and 1.0 minimum wages; proportion of heads of households with <1 year of schooling; proportion of households with 10 or more residents; proportion of rented households; proportion of population aged between 10 and 14 years with no schooling
8	Bratberg, E., Gjesdal, S., & Mæland, J. G. (2009). Sickness absence with psychiatric diagnoses: Individual and contextual predictors of permanent disability. Health and Place, 15(1), 308-314.	NOR	county	deciles	x	x													x		x			x	[illegible]
9	Buckeridge, D., Glazier, R., Harvey, B. J., Escobar, M., Amrhein, C., & Frank, J. (2002). A study of the relationship between vehicle emissions and respiratory health in an urban area. Geographical and Environmental Modelling, 2(1), 23-42.	CAN	Census Enumeration Area	Summed standardized rates	x		x		x	x		x	x	x		x		x	x			x			[illegible]
10	Butler, D. C., Petterson, S., Bazemore, A., & Douglas, K. A. (2010). Use of measures of socioeconomic deprivation in planning primary health care workforce and defining health care need in Australia. Australian Journal of Rural Health, 18(5), 199-204.	AUS	small area level	FA	x			x											x	x					[illegible]
11	Butler, D. C., Petterson, S., Phillips, R. L., & Bazemore, A. W. (2013). Measures of social deprivation that predict health care access and need within a rational area of primary care service delivery. Health Services Research, 48(2 PART1), 539-559.	USA	PCSA	FA	x	x			x	x		x	x	x		x		x	x	x		x			[illegible]
12	Cabrera-Barona, P., Murphy, T., Kienberger, S., & Blaschke, T. (2015). A multi-criteria spatial deprivation index to support health inequality analyses. International Journal of Health Geographics, 14(1).	ECU	census blocks	VIF; AHP					x	x						x		x	x	x				x	[illegible]
13	Cabrera-Barona, P., Wei, C., & Hagenlocher, M. (2016). Multiscale evaluation of an urban deprivation index: Implications for quality of life and healthcare accessibility planning. Applied Geography, 70, 1-10.	ECU	census block, tract	PCA (VIF)					x	x		x		x		x		x	x	x		x		x	[illegible]
14	Carstairs, V. (1981). Multiple Deprivation and Health State. Journal of Public Health (United Kingdom), 3(1), 4-13.	UK	ward	PCA	x	x						x		x		x		x	x	x		x			[illegible]
15	Carstairs, V., & Morris, R. (1991). Deprivation and Health in Scotland. Aberdeen University Press.	UK	Ward	PCA								x		x		x		x	x	x		x			[illegible]
16	Chan, E., Serrano, J., Chen, L., Stieb, D. M., Jerrett, M., & Osornio-Vargas, A. (2015). Development of a Canadian socioeconomic status index for the study of health outcomes related to environmental pollution. BMC Public Health, 15(1).	CAN	Census Dissemination Areas	PCA	x	x	x		x	x		x	x	x		x		x	x	x		x			[illegible]
17	Chiavarini, M., Lanari, D., Minelli, L., & Salmasi, L. (2014). Socio-demographic determinants and access to prenatal care in Italy. BMC Health Services Research, 14(1).	ITA	Municipality	Standardization					x	x		x	x	x		x		x	x			x			[illegible]
18	Choi, M. H., Cheong, K. S., Cho, B. M., Hwang, I. K., Kim, C. H., Kim, M. H., . . . Yoon, T. H. (2011). Deprivation and mortality at the town level in Busan, Korea: An ecological study. Journal of Preventive Medicine and Public Health, 44(6), 242-248.	KOR	town	PCA					x	x		x	x			x		x	x	x		x	x		[illegible]
19	Choi, Y. H., & Lee, S. G. (2011). Does regional socioeconomic context affect the dental caries experience? A multilevel study of Korean adults. European Journal of Oral Sciences, 119(4), 294-300.	KOR	Admin districts	FA								x	x						x	x			x	x	[illegible]
20	Cocheci, R. M. (2015). Individualisation of deprived areas in Romania's south West Region. Journal of Urban and Regional Analysis, 7(2), 161-176.	ROM	LAU2	PCA	x		x					x	x	x		x		x	x	x			x	x	[illegible]
21	Córdoba-Doña, J. A., Novalbos-Ruiz, J. P., Suárez-Farfante, J., Andérica-Frías, G., & Escolar-Pujolar, A. (2012). Social inequalities in HIV-TB and non-HIV-TB patients in two urban areas in southern Spain: Multilevel analysis. International Journal of Tuberculosis and Lung Disease, 16(3), 342-347.	ESP	Tract	Unclear					x	x						x	x	x							[illegible]
22	Coulon, S. J., Velasco-Gonzalez, C., Scribner, R., Park, C. L., Gomez, R., Vargas, A., Hempe, J. M. (2017). Racial differences in neighborhood disadvantage, inflammation and metabolic control in black and white pediatric type 1 diabetes patients. Pediatric Diabetes, 18(2), 120-127.	USA	Tract	FA	x	x										x		x	x			x	x		[illegible]
23	d'Orsi, E., Carvalho, M. S., & Cruz, O. G. (2005). Similarity between neonatal profile and socioeconomic index: a spatial approach. Cadernos de saúde pública / Ministério da Saúde, Fundação Oswaldo Cruz, Escola Nacional de Saúde Pública, 21(3), 786-794.	BRA	Neighborhood	K means clustering	x		x					x	x						x	x					[illegible]
24	Darden, J. T., & Kamel, S. M. (2000). Spatial and socioeconomic analysis of Latin Americans and whites in the Toronto CMA. Journal of Developing Societies, 16(2), 245-270.	CAN	Tract	Summed scores	x	x	x		x	x		x	x		x	x	x	x							[illegible]
25	Department for Communities and Local Government(2015). English indices of deprivation 2015. Retrieved from https://www.gov.uk/government/statistics/english-indices-of-deprivation-2015.	UK	LSOA	FA	x	x			x	x	x	x		x		x		x	x	x	x			x	[illegible]
26	Diez Roux, A. V., Merkin, S. S., Arnett, D., Chambless, L., Massing, M., Nieto, F. J., . . . Watson, R. L. (2001). Neighborhood of residence and incidence of coronary heart disease. New England Journal of Medicine, 345(2), 99-106.	USA	Block Group	Z-scores	x	x	x		x	x		x			x	x	x								[illegible]
27	Dubowitz, T., Heron, M., Basurto-Davila, R., Bird, C. E., Lurie, N., & Escarce, J. J. (2011). Racial/Ethnic Differences in US Health Behaviors: A Decomposition Analysis. American Journal of Health Behavior, 35(3).	USA	census tract	FA	x	x	x		x	x						x		x	x			x			[illegible]
28	Dumedah, G., Schuurman, N., & Yang, W. (2008). Minimizing effects of scale distortion for spatially grouped census data using rough sets. Journal of Geographical Systems, 10(1), 47-69.	CAN	census tract	rough sets	x	x	x		x	x		x			x	x		x							[illegible]
29	Earnest, A., Ong, M. E. H., Shahidah, N., Chan, A., Wah, W., & Thumboo, J. (2015). Derivation of indices of socioeconomic status for health services research in Asia. Preventive Medicine Reports, 2, 326-332.	SIN	planning areas	PCA	x		x	x	x	x	x	x		x		x	x	x							[illegible]
30	Eibner, C., & Sturm, R. (2006). US-based indices of area-level deprivation: Results from HealthCare for Communities. Social Science and Medicine, 62(2), 348-359.	USA	tract	FA	x	x	x		x	x		x		x		x		x	x	x		x	x		[illegible]
31	Esparza, A. X., Waldorf, B. S., & Chavez, J. (2004). Localized effects of globalization: The case of ciudad juárez, chihuahua, mexico. Urban Geography, 25(2), 120-138.	MEX	Geostatistical Districts	PCA					x		x	x		x					x	x			x		[illegible]
32	Scottish Executive(2004). Scottish Index of Multiple Deprivation 2004: Summary Technical Report. Edinburgh: Scottish Executive.	UK	Census Output Area	FA	x	x			x	x						x		x	x	x	x			x	[illegible]
33	Exeter, D. J., Zhao, J., Browne, M., & Lee, A. C. (2016). Towards a new Index of Multiple Area-Level Deprivation for Auckland, New Zealand. New Zealand Geographer, 72(2), 92-106.	NZ	Lower Zone	FA	x	x			x	x		x		x		x		x	x	x	x			x	[illegible]
34	Faria, R., & Santana, P. (2014). Regional inequalities in infant mortality and Primary Health Care in the State of Minas Gerais, Brazil. Espace populations societes, 2014(2-3).	BRA	Admin Microregions	Standardization	x		x		x		x								x	x					[illegible]
35	Field, K. (2000). Measuring the need for primary health care: An index of relative disadvantage. Applied Geography, 20(4), 305-332.	UK	Enumeration District	PCA					x	x		x		x		x	x	x	x	x		x	x		[illegible]
36	Foote, N., & Walter, R. (2016). Neighborhood and socioeconomic change in emerging megapolitan nodes: tracking shifting social geographies in three rapidly growing United States metropolitan areas, 1980–2010. Urban Geography, 1-28.	USA	Tract	PCA	x	x	x		x	x						x		x							[illegible]
37	Frohlich, N., & Mustard, C. (1996). A regional comparison of socioeconomic and health indices in a Canadian province. Social Science and Medicine, 42(9), 1273-1281.	CAN	Municipality	Step-wise Linear Regression					x	x		x			x	x		x	x			x			[illegible]
38	Fukuda, Y., Nakamura, K., & Takano, T. (2007). Higher mortality in areas of lower socioeconomic position measured by a single index of deprivation in Japan. Public Health, 121(3), 163-173.	JAP	Municipality	PCA	x	x	x		x	x		x	x	x		x		x	x			x			[illegible]
39	Godley, J., Haines, V. A., Hawe, P., & Shiell, A. (2010). Small area contextual effects on self-reported health: Evidence from riverside, calgary. BMC Public Health, 10.	CAN	Dissemination Area	FA	x		x		x	x									x			x			[illegible]
40	Gotsens, M., Marí-Dell'Olmo, M., Martínez-Beneito, M. A., Pérez, K., Pasarín, M. I., Daponte, A., Borrell, C. (2011). Socio-economic inequalities in mortality due to injuries in small areas of ten cities in Spain (MEDEA Project). Accident Analysis and Prevention, 43(5), 1802-1810.	ESP	Tract	PCA					x	x						x	x	x							[illegible]
41	Gould, A. C., Apparicio, P., & Cloutier, M. S. (2012). Classifying neighbourhoods by level of access to stores selling fresh fruit and vegetables and groceries: Identifying problematic areas in the city of Gatineau, Quebec. Canadian Journal of Public Health, 103(6), 433-437.	CAN	Dissemination Area	Summed	x		x		x	x						x		x	x			x	x		[illegible]
42	Guillaume, E., Pornet, C., Dejardin, O., Launay, L., Lillini, R., Vercelli, M., Launoy, G. (2016). Development of a cross-cultural deprivation index in five European countries. Journal of Epidemiology and Community Health, 70(5), 493-499.	EU	Municipality - Tract - LSOA	Unclear					x	x		x	x	x		x	x	x	x	x	x	x	x		[illegible]
43	Havard, S., Deguen, S., Bodin, J., Louis, K., Laurent, O., & Bard, D. (2008). A small-area index of socioeconomic deprivation to capture health inequalities in France. Social Science and Medicine, 67(12), 2007-2016.	FRA	Block	PCA	x		x		x	x		x	x			x	x	x	x	x		x			[illegible]
44	Holmqvist, B. M., Lofman, O., & Samuelsson, U. (2008). A low incidence of Type 1 diabetes between 1977 and 2001 in south-eastern Sweden in areas with high population density and which are more deprived. Diabetic Medicine, 25(3), 255-260.	SWE	200m x 200m Square	logged chi-square	x	x	x		x	x						x		x							[illegible]
45	Ivaldi, E., & Testi, A. (2011). Genoa index of deprivation (GDI): An index of material deprivation for geographical areas. Social Indicators: Statistics, Trends and Policy Development (pp. 75-97).	ITA	Urban Units	Box Cox					x	x		x	x	x		x		x							[illegible]
46	Jamal, H., Khan, A. J., Toor, I. A., & Amir, N. (2003). Mapping the spatial deprivation of Pakistan. Pakistan Development Review, 42(2), 91-111.	PAK	Districts	PCA					x	x	x	x	x	x		x	x	x	x	x					[illegible]
47	Jarman, B. (1983). Identification of underprivileged areas. British Medical Journal, 286(6379), 1705-1709.	UK	Ward	Normalization								x		x					x	x		x	x	x	[illegible]
48	Juhász, A., Nagy, C., Páldy, A., & Beale, L. (2010). Development of a Deprivation Index and its relation to premature mortality due to diseases of the circulatory system in Hungary, 1998-2004. Social Science and Medicine, 70(9), 1342-1349.	HUN	Municipality	PCA	x		x		x	x		x		x		x		x	x	x		x			[illegible]
49	Kaufman, J. S., Alonso, F. T., & Pino, P. (2008). Multi-level modeling of social factors and preterm delivery in Santiago de Chile. BMC Pregnancy and Childbirth, 8.	CHILE	Census Districts	PCA					x	x						x	x	x	x	x		x			[illegible]
50	Kearns, A., Gibb, K., & Mackay, D. (2000). Area deprivation in Scotland: A new assessment. Urban Studies, 37(9), 1535-1559.	UK	Postcode	PCA	x	x			x	x		x		x		x		x	x	x					[illegible]
51	Krieger, N., Chen, J. T., Waterman, P. D., Soobader, M. J., Subramanian, S. V., & Carson, R. (2002). Geocoding and monitoring of US socioeconomic inequalities in mortality and cancer incidence: Does the choice of area-based measure and geographic level matter? The public health disparities geocoding project. American Journal of Epidemiology, 156(5), 471-482.	USA	census tract	FA	x	x	x	x	x	x		x		x	x	x	x	x							[illegible]
52	Lalloué, B., Monnez, J. M., Padilla, C., Kihal, W., Le Meur, N., Zmirou-Navier, D., & Deguen, S. (2013). A statistical procedure to create a neighborhood socioeconomic index for health inequalities analysis. International Journal for Equity in Health, 12(1).	FRA	Block Group	PCA	x		x					x	x	x		x	x	x	x	x		x	x		[illegible]
53	Langlois, A., & Kitchen, P. (2001). Identifying and measuring dimensions of urban deprivation in Montreal: An analysis of the 1996 census data. Urban Studies, 38(1), 119-139.	CAN	census tract	PCA	x	x	x		x	x		x	x		x	x		x	x	x		x	x		[illegible]
54	Law, G. R., Smith, A. G., & Roman, E. (2002). The importance of full participation: Lessons from a national case-control study. British Journal of Cancer, 86(3), 350-355.	UK	Enumeration District	Standardization								x	x			x		x	x	x					[illegible]
55	Li, H., & Liu, Y. (2016). Neighborhood socioeconomic disadvantage and urban public green spaces availability: A localized modeling approach to inform land use policy. Land Use Policy, 57, 470-478.	CHN	Districts	PCA	x	x			x	x						x	x	x	x	x			x		[illegible]
56	Lian, M., Pérez, M., Liu, Y., Schootman, M., Frisse, A., Foldes, E., & Jeffe, D. B. (2014). Neighborhood socioeconomic deprivation, tumor subtypes, and causes of death after non-metastatic invasive breast cancer diagnosis: a multilevel competing-risk analysis. Breast Cancer Research and Treatment, 147(3), 661-670.	USA	census tract	PCA	x		x									x		x	x	x		x	x		[illegible]
57	Lisabeth, L. D., Diez Roux, A. V., Escobar, J. D., Smith, M. A., & Morgenstern, L. B. (2007). Neighborhood environment and risk of ischemic stroke: The Brain Attack Surveillance in Corpus Christi (BASIC) Project. American Journal of Epidemiology, 165(3), 279-287.	USA	census tract	Standardization	x	x	x		x	x		x			x	x	x								[illegible]
58	López-De Fede, A., Stewart, J. E., Hardin, J. W., & Mayfield-Smith, K. (2016). Comparison of small-area deprivation measures as predictors of chronic disease burden in a low-income population. International Journal for Equity in Health, 15(1).	USA	ZCTA	Z-scores	x	x	x		x	x		x	x			x	x	x	x	x		x		x	[illegible]
59	Lupo, P. J., Danysh, H. E., Symanski, E., Langlois, P. H., Cai, Y., & Swartz, M. D. (2015). Neighborhood-based socioeconomic position and risk of oral clefts among offspring. American Journal of Public Health, 105(12), 2518-2525.	USA	census tract	PCA	x	x			x	x		x	x	x		x	x	x							[illegible]
60	Mariapun, J., Hairi, N. N., & Ng, C. W. (2016). Are the poor dying younger in Malaysia? An examination of the socioeconomic gradient in mortality. PLoS ONE, 11(6).	MAL	Districts	PCA					x	x		x	x			x		x	x	x		x			[illegible]
61	Marimuthu, S., & Sarvalingam, A. (2010). Human Deprivation Index: A Measure of Multidimensional Poverty. Retrieved from	IND		Weights	x	x			x		x													x	[illegible]
62	Matheson, F. I., Dunn, J. R., Smith, K. L., Moineddin, R., & Glazier, R. H. (2012). Development of the Canadian Marginalization Index: A New Tool for the Study of Inequality. Canadian Journal of Public Health, 103(Suppl.2), S12-S16.	CAN	DA	FA					x	x		x	x	x		x		x	x	x		x	x		[illegible]
63	McCrone, P., & Jacobson, B. (2004). Indicators of mental health activity in London: Adjusting for sociodemographic need. The Health Well.	UK	Local Authority	FA					x	x						x		x	x		x		x	x	[illegible]
64	McCrone, P., Thornicroft, G., Boyle, S., Knapp, M., & Aziz, F. (2006). The development of a Local Index of Need (LIN) and its use to explain variations in social services expenditure on mental health care in England. Health and Social Care in the Community, 14(3), 254-263.	UK	Local Authority	PCA					x	x		x		x		x	x		x	x	x	x	x	x	[illegible]
65	McInerney, M., Csizmadi, I., Friedenreich, C. M., Uribe, F. A., Nettel-Aguirre, A., McLaren, L. McCormack, G. R. (2016). Associations between the neighbourhood food environment, neighbourhood socioeconomic status, and diet quality: An observational study. BMC Public Health, 16(1).	CAN	DA	PCA	x		x		x	x		x	x		x	x		x	x			x			[illegible]
66	McIntyre, D., Muirhead, D., & Gilson, L. (2002). Geographic patterns of deprivation in South Africa: Informing health equity analyses and public resource allocation strategies. Health Policy and Planning, 17(SUPPL. 1), 30-39.	RSA	Magisterial Districts	PCA					x	x						x		x	x	x		x	x		[illegible]
67	McManus, B. M., Robert, S. A., Albanese, A., Sadek-Badawi, M., & Palta, M. (2011). Relationship between neighborhood disadvantage and social function of Wisconsin 2- and 3-year-olds born at very low birth weight. Archives of Pediatrics and Adolescent Medicine, 165(2), 119-125.	USA	census tract	PCA	x	x			x	x						x		x							[illegible]
68	McNamara, J., Cassells, R., Wicks, P., & Vidyattama, Y. (2010). Children in housing disadvantage in Australia: Development of a summary small area index. Housing Studies, 25(5), 625-646.	AUS	Statistical Local Area	Standardization								x	x	x											[illegible]
69	Morton, W. E., Horton, H. B., & Baker, H. W. (1979). Effects of Socioeconomic Status on Incidences of Three Sexually Transmitted Diseases. Sexually Transmitted Diseases, 6(3), 206-210.	USA	census tract	n/a	x	x	x		x	x		x		x											[illegible]
70	Meijer, M., Engholm, G., Grittner, U., & Bloomfield, K. (2013). A socioeconomic deprivation index for small areas in Denmark. Scandinavian Journal of Public Health, 41(6), 560-569. doi:10.1177/1403494813483937	DEN	Parish	PCA	x		x		x	x		x	x	x		x	x	x	x	x	x	x			[illegible]
71	Mustard, C. A., & Frohlich, N. (1995). Socioeconomic status and the health of the population. Medical Care, 33(12), DS43-DS54.	CAN	Municipality	Step-wise Linear Regression					x	x		x			x	x		x	x			x			[illegible]
72	Messer, L. C., Laraia, B. A., Kaufman, J. S., Eyster, J., Holzman, C., Culhane, J., . . . O'Campo, P. (2006). The development of a standardized neighborhood deprivation index. Journal of Urban Health, 83(6), 1041-1062.	USA	census tract	PCA	x	x	x									x	x	x							[illegible]
73	Nyamenga, T., Trepka, M. J., Lieb, S., & Maddox, L. M. (2013). Measuring socioeconomic inequality in the incidence of AIDS: Rural-Urban considerations. AIDS and Behavior, 17(2), 700-709.	USA	ZCTA	PCA	x	x	x	x	x	x		x	x	x	x	x	x	x	x	x					[illegible]

74	Noble, M., Barnes, H., Wright, G., McLennan, D., Avenell, D., Whitworth, A., & Roberts, B. (2009). The South African Index of Multiple Deprivation 2001 at Datazone Level. Pretoria: Republic of South Africa.	RSA	Data Zone	Shrinkage	[illegible]
75	Noble, M., Sin Yi, C., Smith, G., & Smith, T. (1995). Using census data to predict income support dependency. Policy & Politics, 23(4), 327-333.	UK	Enumeration District	Linear Regression	[illegible]
76	Northern Ireland Statistics and Research Agency(2010). Northern Ireland multiple deprivation measure 2010. Retrieved from http://www.nisra.gov.uk/deprivation/nimdm_2010.htm.	UK	Super Output Area	Ranks	[illegible]
77	Ocaña-Riola, R., Saurina, C., Fernández-Ajuria, A., Lertxundi, A., Sánchez-Cantalejo, C., Saez, M., Benach, J. (2008). Area deprivation and mortality in the provincial capital cities of Andalusia and Catalonia (Spain). Journal of Epidemiology and Community Health, 62(2), 147-152.	ESP	Census Section	PCA	[illegible]
78	Ogneva-Himmelberger, Y., Rakshit, R., & Pearsall, H. (2013). Examining the Impact of Environmental Factors on Quality of Life Across Massachusetts. Professional Geographer, 65(2), 187-204.	USA	Block Group	PCA	[illegible]
79	Pampalon, R., & Raymond, G. (2000). A deprivation index for health and welfare planning in Quebec. Chronic Diseases in Canada, 21(3), 104-113.	CAN	Enumeration District	FA	[illegible]
80	Panczak, R., Galobardes, B., Voorpostel, M., Spoerri, A., Zwahlen, M., & Egger, M. (2012). A Swiss neighbourhood index of socioeconomic position: Development and association with mortality. Journal of Epidemiology and Community Health, 66(12), 1129-1136.	SWI	Neighborhood	PCA	[illegible]
81	Patel, S. A., Sherman, S. G., Khatry, S. K., LeClerq, S. C., Katz, J., Tielsch, J. M., & Christian, P. (2016). An Index of Community-Level Socioeconomic Composition for Global Health Research. Social Indicators Research, 129(2), 639-658.	NEP	Village Development Committee	PCA	[illegible]
82	Pearce, J. R., Richardson, E. A., Mitchell, R. J., & Shortt, N. K. (2011). Environmental justice and health: A study of multiple environmental deprivation and geographical inequalities in health in New Zealand. Social Science and Medicine, 73(3), 410-420.	NZ	Census Area Unit	Standardization	[illegible]
83	Pink, B. (2011). Socio-Economic Indexes for Areas (SEIFA). Canberra, AUS: Commonwealth of Australia.	AUS	SA1	PCA	[illegible]
84	Platt, A. B., Field, S. H., Asch, D. A., Chen, Z., Patel, N. P., Gupta, R., . . . Kuna, S. T. (2009). Neighborhood of residence is associated with daily adherence to CPAP therapy. Sleep, 32(6), 799-806.	USA	Block Group	PCA	[illegible]
85	Pornet, C., Delpierre, C., Dejardin, O., Grosclaude, P., Launay, L., Guittet, L., Launoy, G. (2012). Construction of an adaptable European transnational ecological deprivation index: The French version. Journal of Epidemiology and Community Health, 66(11), 982-989.	FRA	Blocks	Logistic Regression	[illegible]
86	Pratschke, J., & Haase, T. (2007). Measurement of social disadvantage and its spatial articulation in the Republic of Ireland. Regional Studies, 41(6), 719-734.	IRE	Census Tract	Structural Equations Model	[illegible]
87	Rey, G., Jougla, E., Fouillet, A., & Hémon, D. (2009). Ecological association between a deprivation index and mortality in France over the period 1997 - 2001: Variations with spatial scale, degree of urbanicity, age, gender and cause of death. BMC Public Health, 9.	FRA	Commune	PCA	[illegible]
88	Ribeiro, A. I., Mayer, A., Miranda, A., & Pina, M. D. F. D. (2017). The portuguese version of the european deprivation index: An instrument to study health inequalities. Acta Medica Portuguesa, 30(1), 17-25.	POR	Tract Block Group	Regression	[illegible]
89	Richardson, E. A., Mitchell, R., Shortt, N. K., Pearce, J., & Dawson, T. P. (2010). Developing summary measures of health-related multiple physical environmental deprivation for epidemiological research. Environment and Planning A, 42(7), 1650-1668.	UK	Ward	Standardization	[illegible]
90	Richardson, E. A., Pearce, J., Mitchell, R., & Shortt, N. K. (2013). A Regional Measure of Neighborhood Multiple Environmental Deprivation: Relationships with Health and Health Inequalities. Professional Geographer, 65(1), 153-170.	UK	Data Zone	FA	[illegible]
91	Roblin, D. W. (2013). Validation of a Neighborhood SES Index in a Managed Care Organization. Medical Care, 51(1), e1-e8.	USA	Block Group	PCA	[illegible]
92	Rodero-Cosano, M. L., Garcia-Alonso, C. R., & Salinas-Pérez, J. A. (2014). A deprivation analysis for Andalusia (Spain): An approach based on structural equations. Social Indicators Research, 115(2), 751-765.	ESP	Municipality	Structural Equations Model	[illegible]
93	Salcedo, N., Saez, M., Bragulat, B., & Saurina, C. (2012). Does the effect of gender modify the relationship between deprivation and mortality? BMC Public Health, 12(1).	ESP	Tract	Pena Distance	[illegible]
94	Salinas, J. J., Shah, M., Abdelbary, B., Gay, J. L., & Sexton, K. (2012). Application of a novel method for assessing cumulative risk burden by county. International Journal of Environmental Research and Public Health, 9(5), 1820-1835.	USA	County	FA	[illegible]
95	Sánchez-Cantalejo, C., Ocana-Riola, R., & Fernández-Ajuria, A. (2008). Deprivation index for small areas in Spain. Social Indicators Research, 89(2), 259-273.	ESP	Municipality	PCA	[illegible]
96	Schneiders, J., Drukker, M., Van Der Ende, J., Verhulst, F. C., Van Os, J., & Nicolson, N. A. (2003). Neighbourhood socioeconomic disadvantage and behavioural problems from late childhood into early adolescence. Journal of Epidemiology and Community Health, 57(9), 699-703.	NED	Neighborhood	PCA	[illegible]
97	Sealy-Jefferson, S., Slaughter-Acey, J., Caldwell, C. H., Kwarteng, J., & Misra, D. P. (2016). Neighborhood disadvantage and preterm delivery in Urban African Americans: The moderating role of religious coping. SSM - Population Health, 2, 656-661.	USA	Block Group	PCA	[illegible]
98	Singh, G. K. (2003). Area Deprivation and Widening Inequalities in US Mortality, 1969-1998. American Journal of Public Health, 93(7), 1137-1143.	USA	County, Zip Code	PCA	[illegible]
99	Singh, G. K., Miller, B. A., & Hankey, B. F. (2002). Changing area socioeconomic patterns in U.S. cancer mortality, 1950-1998: Part II - Lung and colorectal cancers. Journal of the National Cancer Institute, 94(12), 916-925.	USA	Zip Code, Census Tract	PCA	[illegible]
100	Singh, G. K., & Siahpush, M. (2006). Widening socioeconomic inequalities in US life expectancy, 1980-2000. International Journal of Epidemiology, 35(4), 969-979.	USA	Zip Code, Census Tract	PCA	[illegible]
101	Šlachtová, H., Tomášková, H., Šplíchalová, A., Polaufová, P., & Fejtková, P. (2009). Czech socio-economic deprivation index and its correlation with mortality data. International Journal of Public Health, 54(4), 267-273.	CZE	Districts	Standardization	[illegible]
102	Sloggett, A., & Joshi, H. (1998). Indicators of deprivation in people and places: longitudinal perspectives. Environment and Planning A, 30(6), 1055-1076.	UK	Ward	Z-scores	[illegible]
103	South, S. J. (2001). The geographic context of divorce: Do neighborhoods matter? Journal of Marriage and Family, 63(3), 755-766.	USA	census tract	Standardization	[illegible]
104	Souza, W. V., Ximenes, R., Albuquerque, M. F. M., Lapa, T. M., Portugal, J. L., Lima, M. L. C., & Martelli, C. M. T. (2000). The use of socioeconomic factors in mapping tuberculosis risk areas in a city of northeastern Brazil. Revista Panamericana de Salud Publica/Pan American Journal of Public Health, 8(6), 403-410.	BRA	census tract	PCA	[illegible]
105	Spadea, T., Zengarini, N., Kunst, A., Zanetti, R., Rosso, S., & Costa, G. (2010). Cancer risk in relationship to different indicators of adult socioeconomic position in Turin, Italy. Cancer Causes and Control, 21(7), 1117-1130.	ITA	census tract	Standardization	[illegible]
106	Statistics for Wales, (2014). Welsh Index of Multiple Deprivation (WIMD) 2014. Crown Copyright.	UK	LSOA	FA	[illegible]
107	Su, S., Gong, Y., Tan, B., Pi, J., Weng, M., & Cai, Z. (2016). Area Social Deprivation and Public Health: Analyzing the Spatial Non-stationary Associations Using Geographically Weighed Regression. Social Indicators Research, 1-14.	CHN	Districts	PCA	[illegible]
108	Sundquist, K., Malmström, M., Johansson, S. E., & Sundquist, J. (2003). Care need index, a useful tool for the distribution of primary health care resources. Journal of Epidemiology and Community Health, 57(5), 347-352.	SWE	Small Area Marked Statistics	Weights	[illegible]
109	Tanton, R., Harding, A., Daly, A., McNamara, J., & Yap, M. (2010). Australian children at risk of social exclusion: A spatial index for gauging relative disadvantage. Population, Space and Place, 16(2), 135-150.	AUS	Statistical Local Area	PCA	[illegible]
110	Terashima, M., Rainham, D. G. C., & Levy, A. R. (2013). Should we enhance the commonly used deprivation index for a regional context? Canadian Journal of Public Health, 104(4), e311-e316.	CAN	Communities	FA	[illegible]
111	Testi, A., & Ivaldi, E. (2009). Material versus social deprivation and health: A case study of an urban area. European Journal of Health Economics, 10(3), 323-328.	ITA	Urban Units	FA	[illegible]
112	Tompkins, J. W., Luginaah, I. N., Booth, G. L., & Harris, S. B. (2010). The geography of diabetes in London, Canada: The need for local level policy for prevention and management. International Journal of Environmental Research and Public Health, 7(5), 2407-2422.	CAN	Census Tract	PCA	[illegible]
113	Torres-Cintrón, M., Ortiz, A. P., Ortiz-Ortiz, K. J., Figueroa-Vallés, N. R., Pérez-Irizarry, J., Díaz-Medina, G., . . . Suárez-Pérez, E. (2012). Using a socioeconomic position index to assess disparities in cancer incidence and mortality, Puerto Rico, 1995-2004. Preventing Chronic Disease, 9(1).	UERTO RIC	Municipality	PCA	[illegible]
114	Townsend, P., Phillimore, P., & Beattie, A. (1988). Health and Deprivation: Inequality and the North. Croom Helm, AUS: Croom Helm.	UK	Wards	PCA	[illegible]
115	Trinity College Dublin, D. o. H. a. G. P., Small Area Health Research Unit. (1997). A National Deprivation Index for Health and Health Services Research. Retrieved from Dublin	IRE	Ward	PCA	[illegible]
116	Tu, W., Tu, J., & Tedders, S. (2014). Estimating neighbourhood-level socio-economic effect on preterm births using a multilevel approach: A case study in Georgia, USA. Annals of GIS, 20(3), 181-191.	USA	Census Tract	PCA	[illegible]
117	Vasquez, A., Cabieses, B., & Tunstall, H. (2016). Where are socioeconomically deprived immigrants located in Chile? A spatial analysis of census data using an index of multiple deprivation from the last three decades (1992-2012). PLoS ONE, 11(1).	CHE	County	Normalization	[illegible]
118	Wan, C., & Su, S. (2016). Neighborhood housing deprivation and public health: Theoretical linkage, empirical evidence, and implications for urban planning. Habitat International, 57, 11-23.	CHN	Districts	PCA	[illegible]
119	Wan, C., & Su, S. (2017). China's social deprivation: Measurement, spatiotemporal pattern and urban applications. Habitat International, 62, 22-42.	CHN	Prefecture City	PCA	[illegible]
120	Wang, F., & Arnold, M. T. (2008). Localized income inequality, concentrated disadvantage and homicide. Applied Geography, 28(4), 259-270.	USA	census tract	PCA	[illegible]
121	Weng, M., Pi, J., Tan, B., Su, S., & Cai, Z. (2016). Area Deprivation and Liver Cancer Prevalence in Shenzhen, China: A Spatial Approach Based on Social Indicators. Social Indicators Research, 1-16.	CHN	Districts	AHP	[illegible]
122	Windenberger, F., Rican, S., Jougla, E., & Rey, G. (2012). Spatiotemporal association between deprivation and mortality: Trends in France during the nineties. European Journal of Public Health, 22(3), 347-353	FRA	Commune	PCA	[illegible]
123	Winkleby, M., Sundquist, K., & Cubbin, C. (2007). Inequities in CHD Incidence and Case Fatality by Neighborhood Deprivation. American Journal of Preventive Medicine, 32(2), 97-106.	SWE	Small Area Marked Statistics	PCA	[illegible]
124	Witt, W. P., Park, H., Wisk, L. E., Cheng, E. R., Mandell, K., Chatterjee, D., & Zarak, D. (2015). Neighborhood disadvantage, preconception stressful life events, and infant birth weight. American Journal of Public Health, 105(5), 1044-1052.	USA	County	PCA	[illegible]
125	Wong, C. M., Ou, C. Q., Chan, K. P., Chau, Y. K., Thach, T. Q., Yang, L., Lam, T. H. (2008). The effects of air pollution on mortality in socially deprived urban areas in Hong Kong, China. Environmental Health Perspectives, 116(9), 1189-1194.	CHN	TPU	FA	[illegible]
126	Yu, M., Tatalovich, Z., Gibson, J. T., & Cronin, K. A. (2014). Using a composite index of socioeconomic status to investigate health disparities while protecting the confidentiality of cancer registry data. Cancer Causes and Control, 25(1), 81-92.	USA	census tract	FA	[illegible]
127	Yuan, Y., & Wu, F. (2014). The development of the index of multiple deprivations from small-area population census in the city of Guangzhou, PRC. Habitat International, 41, 142-149.	CHN	Committee Areas	FA	[illegible]
128	Yun, J. W., Kim, Y. J., & Son, M. (2016). Regional deprivation index and socioeconomic inequalities related to infant deaths in Korea. Journal of Korean Medical Science, 31(4), 568-578.	KOR	Regions	PCA	[illegible]
129	Zeitlin, J., Combier, E., Levaillant, M., Lasbeur, L., Pilkington, H., Charreire, H., & Rivera, L. (2011). Neighbourhood socio-economic characteristics and the risk of preterm birth for migrant and non-migrant women: A study in a French district. Paediatric and Perinatal Epidemiology, 25(4), 347-356.	FRA	census tract	Standardization	[illegible]
130	Zwanziger, J., & Khan, N. (2008). Safety-net hospitals. Medical Care Research and Review, 65(4), 478-495.	USA	Zip Codes, HSA	Cluster Analysis	[illegible]
