# Supplementary material for: A systematic review of geographic indices of disadvantage with implications for older adults
Source: JCI Insight. 2021 Oct 22;6(20):e141664. doi: 10.1172/jci.insight.141664 (PMC8564893; doi:10.1172/jci.insight.141664)
Supplement: Trial reporting checklists [file jciinsight-6-141664-s072.pdf]

JCI The Journal of Clinical Investigation

Click on boxes to check/uncheck items.

Checklist for submitting a revised Clinical Medicine manuscript

In addition to addressing the items noted in the decision letter regarding your manuscript, ensure that your revised manuscript adheres to the guidelines below. For full submission details, visit the JCI website.

Required files

Manuscript

☑ PDF of a clean version of the entire manuscript, including figures, figure legends, and tables

☑ PDF of a marked-up version of the entire manuscript showing revisions and prefaced by a point-by-point response to reviewer comments

☑ Word or RTF file of all text of the submission, including figure legends, tables, table legends, and references (without figures, images, or point-by-point responses)

☑ Single PDF file of completed ICMJE uniform disclosure forms from all authors

☐ For clinical trials, PDF of the appropriate reporting checklist (CONSORT, STROBE. etc.)

Figures

☑ Journal staff will contact corresponding authors to request publication-quality figures in TIFF format. See detailed instructions for figure preparation.

☐ Recommended: Graphical abstract (details available here)

Supplemental material

☐ Single file containing supplemental material, figures, and modest-sized tables, as:

☐ (a) (if applicable) a PDF highlighting reviewer-requested changes

☐ (b) a clean, publication-quality PDF

☐ Upload any supplemental videos and/or large Excel files separately

Gels

☑ A PDF, PPT, or PPTX file (distinct from any other supplemental material) that shows the entire unedited gel

☑ Clearly indicate which bands were used for the figures

Formatting

☑ Maximum 12,000 words (all text inclusive of title page, full text, references, figure legends, and tables)

☑ Double-spacing throughout, including references and tables; figure legends may be single spaced if necessary to keep a figure and its legend on the same page

☑ All pages numbered

☐ Each section begins on a new page

Abbreviations and acronyms

☐ Standard JCI abbreviations and acronyms used without definition

☐ All other abbreviations and acronyms spelled out at first use in the Abstract and again at first use in the main text (with the abbreviated form appearing in parentheses), and used without definition thereafter

Gene and protein names and symbols

☐ Conform to official NCBI Gene Nomenclature

☐ Presented according to JCI Gene nomenclature and style

Italicization

☑ Generally reserved for gene symbols, genotypes, and species names

☑ Terms such as in vivo, in vitro, etc., are not italicized

Unpublished data, manuscripts in preparation or under review, and personal communications

☑ Cited parenthetically in the text, not as numbered references; e.g., "(Jane L. Doe, UCLA, Los Angeles, California, USA, unpublished observations)"

☑ Written permission to cite unpublished observations of someone outside the author's research team (an email is sufficient) is submitted

Reference citations

☑ Appear in parentheses preceded by a space, e.g., "as described previously (1, 2)"; "several research groups (4–10) have found"

☑ No superscript, bold, italics, etc.

Figure and table callouts

☑ Figures and tables called out in numerical order

☑ "Figure", "Table", "Supplemental Figure", "Supplemental Table", etc., spelled out

☑ Callouts in parentheses (no boldface or italics) unless grammatically part of the sentence: "the levels increased (Figure 5A)"; "data shown in Table 2"

☑ Parts called out as follows: "Figure 1A", "Figure 2, A and B", "Figure 3, B–D"

Manuscript preparation and required reporting

Title page

Manuscript title

☑ Clear, concise, and limited to 15 words, including conjunctions

☑ Refers to the relevant disease or disease model studied

☑ No subtitles, colons, periods, or nonstandard abbreviations

Authors and affiliations

☑ Author names provided in full (for example, "Benita J. Sjögren") and in the appropriate order

☑ No titles, honorifics, degrees, or certifications

☑ Author affiliations correspond to the period when the work was performed

☑ For authors whose affiliation has changed since completion of the work, specify the present affiliation and location below the numbered list

☑ Affiliation footnotes assigned consecutively using superscripted numbers (1, 2, 3, etc.)

☑ Affiliations include departments, institutions, city, state (if applicable), and country (but not mailing addresses or zip/regional codes)

☑ Corresponding author's complete name, address, telephone number (including country code if applicable), and email address

Consortium/study groups shown as authors (e.g., CARDIoGRAM Consortium)

☑ Unless the members of the group appear as authors, each individual member and their affiliation are listed in the supplemental material, under the heading Supplemental Acknowledgments

☐ The following sentence appears in Acknowledgments: "See Supplemental Acknowledgments for details on {name of consortium}."

Conflict-of-interest statement

☑ A statement consistent with the Journal's conflict-of-interest policy is included; if no author has a conflict, state the following: "The authors have declared that no conflict of interest exists."

☑ If patents are involved, the patent or patent application number(s) are provided, and the names of the related authors specified

Abstract

☐ Structured format with the sections Background, Methods, Results, Conclusion, Trial registration, Funding
☑ Maximum 250 words
☑ No references
☑ All nonstandard abbreviations defined at first use

Main text (presented in the following order)

☐ **Introduction**
☐ **Results**
☐ **Discussion**
☐ **Methods**
☑ Demographics reporting:
☐ Inclusive data provided; e.g., do not present values only for male, or only for White, participants, or classify groups of people as “other” without explanation
☐ If this information is not available or is incomplete, briefly explain why
☐ Specify whether the participants or investigators made the classifications
☐ Complete manufacturer name (omit location) provided for each proprietary item used
☐ For animal models, precise genotype, strain, number of backcrosses, sex, age specifed
☐ Description of all antibodies used, including the source and catalog/clone number for commercial antibodies or (reference to) a description of the generation of custom antibodies
☐ Source of all cell lines used is indicated
☐ Data sets for gene expression microarrays, SNP arrays, and high-throughput sequencing studies are deposited in a public repository, and accession number(s) provided in Methods the main text
☐ **Statistics**
☐ Section appears near the end of Methods (before “Study approval”)
☐ The *P* value used to determine significance is specified; e.g., “A *P* value less than 0.05 was considered significant.”
☐ Analysis appropriately corrects for multiple comparisons (more than 2 groups) and for repeated measures (multiple measurements within subjects)
☐ If samples were excluded, a statement describes inclusion/exclusion criteria
☐ **Study approval**
☐ Stand-alone paragraph at the end of Methods
☐ Declaration of approval of human and/or animal studies, specifying the name and location of the appropriate institutional review board(s)
☐ For human studies, a statement indicating receipt of written informed consent from participants and/or their parents/guardians
☐ For use of photographs of patients, a separate statement of written informed consent
☑ **Author contributions**
☑ Contribution of each author (identified by initials) is specified
☑ Grammatically complete sentences
☑ For manuscripts with 2 or more co–first authors, the method used to assign authorship order among these author is stated
☑ **Acknowledgments**
☑ States sources of support in the form of grants, equipment, or drugs
☑ Grant numbers provided as applicable
☑ Other appropriate acknowledgments, such as of colleagues for advice

☐ References

☐ Styled according to Journal reference instructions

☑ Figure legends

☑ Maximum 300 words
☑ Each begins with stand-alone title, irrespective of the individual parts
☑ Figure parts called out in boldface: (**A**), (**B**–**D**), (**C** and **E**)
☑ Symbols and abbreviations introduced in figures are defined
☑ In each figure legend where appropriate, the statistical test(s) used is described
☑ Variance around the mean and statistical analysis not provided for figures representing fewer than 3 independent samples
☑ For figure panels representing multiple experiments, exact number of samples (*n*) is reported
☑ For representative experiments, the number of times the experiment was conducted is reported
☑ For histological panels and insets, scale bars are defined or total original magnification specified in the figure legends

☑ Figures

☑ Prepared according to Journal figure instructions
☑ For clinical trials, the appropriate flow diagram appears as a figure
☑ Parts labeled with capital letters: A, B, C, etc., with no designated subparts
☑ Graphs of quantitative data presented as either dot plots, with average and appropriate error bars indicated; or box-and-whisker plots, with values defined in the legend (bounds of the boxes, lines within the boxes, whiskers, and any outlying values); dynamite plunger plots are not permitted
☑ If lanes in a gel or blot image are spliced together into a composite image, the lanes are separated with a thin vertical line (black on gray background; white on black background); a note in the legend states that the lanes were run on the same gel but were noncontiguous

☑ Tables

☑ Prepared in Word table format (not pasted in from another application)
☑ Self-contained and self-explanatory
☑ Each table fits on a single page and is presented on its own page
☑ Preceded by brief titles
☑ Callouts to footnotes (designated with superscript capital letters) assigned alphabetically row by row
☑ No subparts or subsections (for example, Table 1A and Table 1B)
☑ Column headings in tables apply to all values throughout the column; a new row of column headings may not be introduced within a table
☑ See “Methods” above for demographics reporting

☑ Supplemental material

☑ A single PDF file includes all supplemental material except videos and spreadsheets. See “Methods” above for large data sets
☐ Before submission, carefully review all supplmental files; they will not be checked by a copy editor. The Journal is not responsible for any errors contained in supplemental material.
